# Academic procrastination in university students: Associated factors and impact on academic performance

**DOI:** 10.1192/j.eurpsy.2021.2013

**Published:** 2021-08-13

**Authors:** M. Ben Alaya, U. Ouali, S. Ben Youssef, A. Aissa, F. Nacef

**Affiliations:** 1 Psychiatry A, Razi Hospital, Manouba, Tunisia; 2 Department Psychiatry A, Razi Hospital, Manouba, Tunisia; 3 Nih, National Institute of Health, Tunis, Tunisia

**Keywords:** academic performance, alcohol use, Procrastination, Impulsivity

## Abstract

**Introduction:**

Academic procrastination is a specific sub-type of procrastination, assessing the tendency to delay academic tasks in connection with the preparation of courses or exams.

**Objectives:**

To determine the impact of academic procrastination on studies and academic performances and identify associated factors.

**Methods:**

We conducted a cross-sectional descriptive study in students from three different universities: a medical school, a law school and an engineering school. Socio-demographic, clinical and academic data were collected. Procrastination was assessed using the Academic Procrastination Scale. We further administered the Short Version of the impulsive behaviour scale, the Satisfaction with Life Scale, the Perfectionism scale, and the one item Self-esteem Scale.

**Results:**

Our sample consisted of 1019 students. The mean age was 22 ± 2.25 years, and 62% were females. About one third of study participants used tobacco or alcohol, and 10% used drugs (cannabis or others). We found a significant positive correlation between procrastination and academic failure (r=0.22 p= 0.00) and a negative correlation with academic success (r= -0.27 p=0.00). Multivariate regression analysis showed the following risk factors for academic procrastination: alcohol consumption (ORa= 1.74 [1.14; 2.67]), study field (with reference to medicine: law ORa= 1.50 [1.02; 2.19], engineering studies ORa= 2.01 [1.34; 3.02]), and impulsivity (ORa= 2.11 [1.55; 2.86]).

**Conclusions:**

Academic procrastination has a negative impact on academic achievement and performance. This impact appears to differ depending on the field of study. It also seems closely related to impulsiveness and alcohol use. Our findings might contribute to find new ways of helping students to improve academic performance.

**Disclosure:**

No significant relationships.

